# Supplementary material for: Noise-induced hearing loss delays auditory learning and reduces the influence of acoustic evidence on choice
Source: bioRxiv. 2026 Sep 14:2026.09.10.750711. Preprint. [Version 1] doi: 10.64898/2026.09.10.750711 (PMC13596562; doi:10.64898/2026.09.10.750711)
Supplement: Supplementary file 1 [file NIHPP2026.09.10.750711V1-supplement-1.pdf]

bioRxiv preprint doi: https://doi.org/10.64898/2026.09.10.750711; this version posted September 14, 2026. The copyright holder for this preprint (which was not certified by peer review) is the author/funder, who has granted bioRxiv a license to display the preprint in perpetuity. It is made available under a CC-BY 4.0 International license.

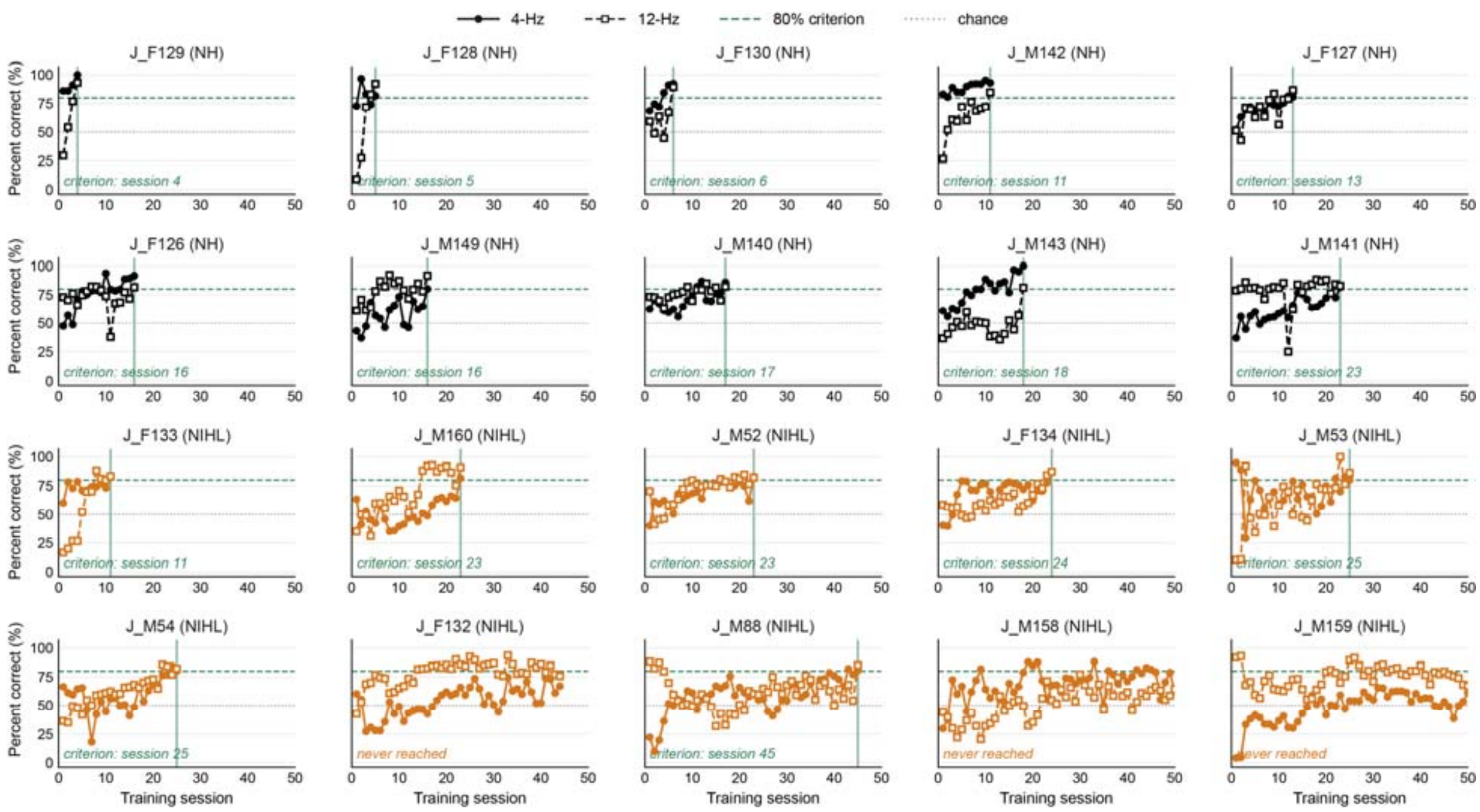

Supplementary Figure S1. Learning curves across training sessions for all animals. Percent correct for the 4-Hz (solid line, filled circles) and 12-Hz (dashed line, open squares) AM rates is shown for all normal-hearing (black) and NIHL (orange) animals. Animals are ordered within each hearing status group by the number of sessions required to reach criterion. The horizontal green dashed line marks the 80% acquisition criterion and the grey dotted line marks chance performance (50%). For animals that reached criterion, the vertical green line indicates the first session in which performance was ≥80% correct for both AM rates. J_F132, J_M158, and J_M159 were discontinued without reaching criterion.

bioRxiv preprint doi: https://doi.org/10.64898/2026.09.10.750711; this version posted September 14, 2026. The copyright holder for this preprint (which was not certified by peer review) is the author/funder, who has granted bioRxiv a license to display the preprint in perpetuity. It is made available under a CC-BY 4.0 International license.

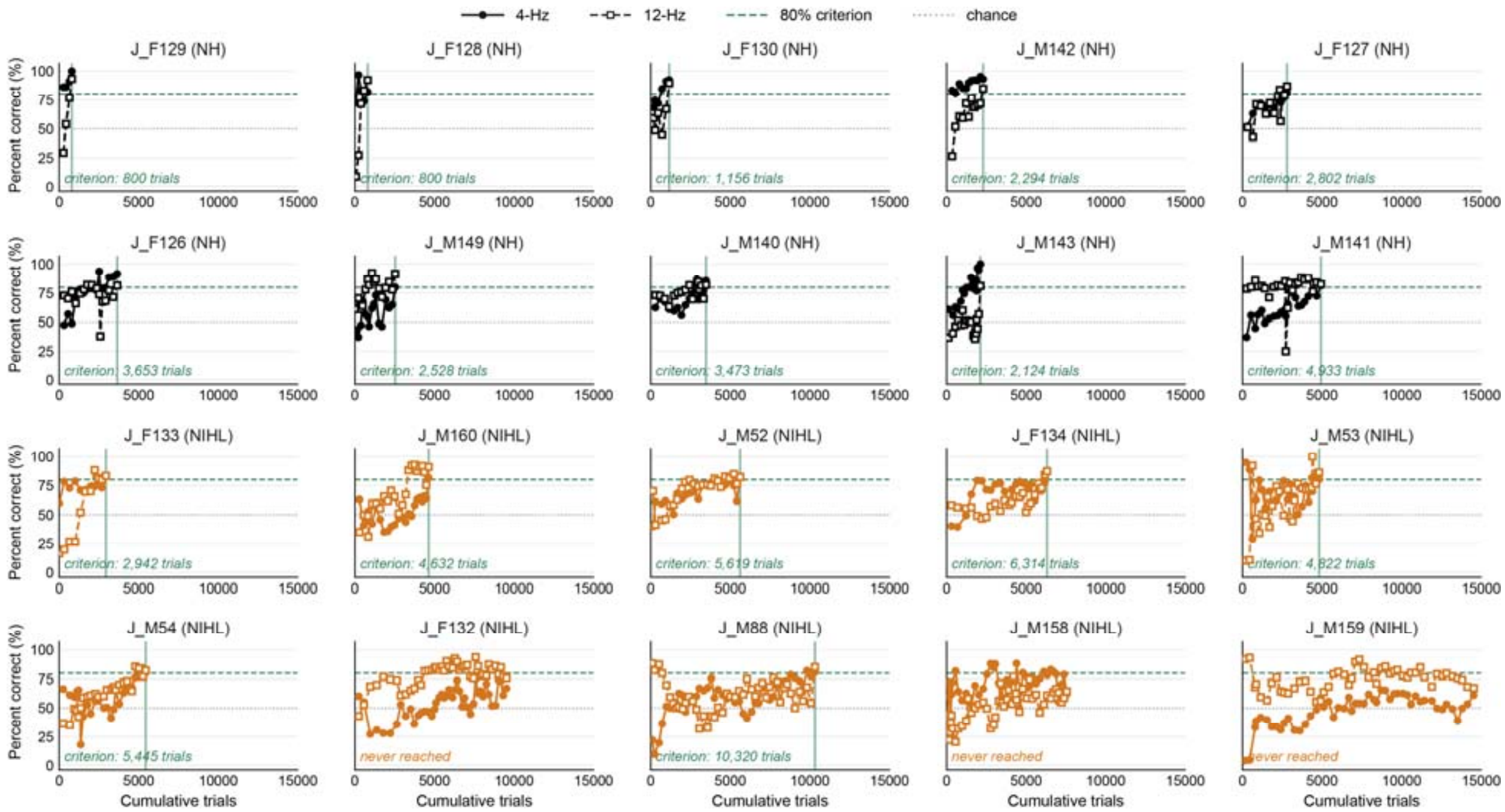

Supplementary Figure S2. Learning curves across cumulative trials for all animals. Percent correct for the 4-Hz (solid line, filled circles) and 12-Hz (dashed line, open squares) AM rates is shown for all normal-hearing (black) and NIHL (orange) animals. Animals are ordered within each hearing status group by the number of sessions required to reach criterion, matching Supplementary Figure 1. The horizontal green dashed line marks the 80% acquisition criterion and the grey dotted line marks chance performance (50%). For animals that reached criterion, the vertical green line indicates the cumulative trial count at the first session in which performance was ≥80% correct for both AM rates. J_F132, J_M158, and J_M159 were discontinued without reaching criterion. N = 10 animals per hearing status group.

bioRxiv preprint doi: https://doi.org/10.64898/2026.09.10.750711; this version posted September 14, 2026. The copyright holder for this preprint (which was not certified by peer review) is the author/funder, who has granted bioRxiv a license to display the preprint in perpetuity. It is made available under a CC-BY 4.0 International license.

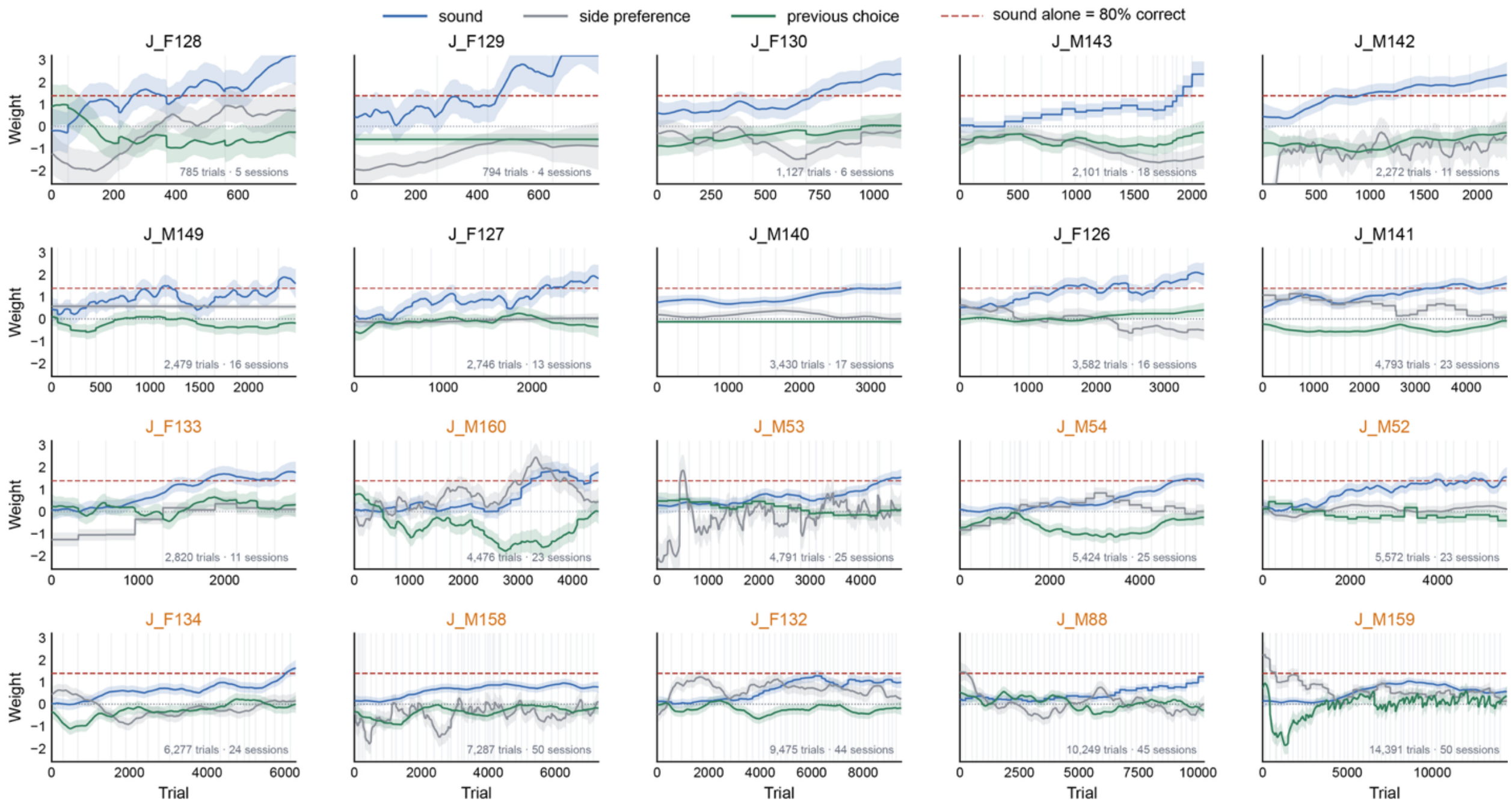

Supplementary Figure S3. Trial-by-trial dynamic logistic regression fits for all animals. Trial-by-trial model weights are shown across the complete training record for all NH (black animal labels) and NIHL animals. Blue lines represent sound weight, gray lines represent side-bias weight, and green lines represent previous-choice weight. Shaded regions indicate ± 2 posterior SD. Vertical gray lines indicate session boundaries. The horizontal red dashed line marks the sound weight at which the sound alone would support 80% correct performance. The total number of trials and training sessions included in each fit is indicated within each panel. Animals are ordered by hearing status group and total number of training trials.
